# A Comparative Cross-Sectional Study Assessing the Psycho-Emotional State of Intensive Care Units’ Physicians and Nurses of COVID-19 Hospitals of a Russian Metropolis

**DOI:** 10.3390/ijerph19031828

**Published:** 2022-02-06

**Authors:** Artem Kashtanov, Ekaterina Molotok, Andrey Yavorovskiy, Alexander Boyarkov, Yuriy Vasil’ev, Ali Alsaegh, Sergey Dydykin, Olesya Kytko, Renata Meylanova, Yulianna Enina, Vasiliy Troitskiy, Marina Kapitonova, Sergey Vaits, Tat’yana Vaits, Rinat Saleev, Gulshat Saleeva, Nail Saleev

**Affiliations:** 1 N.V. Sklifosovskiy Institute of Clinical Medicine, I.M. Sechenov First Moscow State Medical University (Sechenov University), 119991 Moscow, Russia; kashtanovartem001@gmail.com (A.K.); yavor@bk.ru (A.Y.); y_vasiliev@list.ru (Y.V.); dydykin_ss@mail.ru (S.D.); kytkodoc@yandex.ru (O.K.); pheonix75@mail.ru (R.M.); troickii_vasilii@mail.ru (V.T.); 2 Institute of Psychological and Social Work, I.M. Sechenov First Moscow State Medical University (Sechenov University), 119991 Moscow, Russia; katenochek.m@mail.ru; 3 Department of Anaesthesiology and Reanimation, City Clinical Hospital No. 40, Kommunarka, 115516 Moscow, Russia; anestezista86@gmail.com; 4 Department of General Dentistry, Belarusian Medical Academy of Postgraduate Education, 220013 Minsk, Belarus; 5 E.V. Borovsky Institute of Dentistry, I.M. Sechenov First Moscow State Medical University (Sechenov University), 119991 Moscow, Russia; yuliannatr@mail.ru; 6 Faculty of Medicine and Health Sciences, Universiti Malaysia Sarawak (UNIMAS), Kota Samarahan 94300, Sarawak, Malaysia; kmarina@unimas.my; 7 Institute of Medicine, Peoples’ Friendship University of Russia (RUDN University), 117198 Moscow, Russia; swsstom@yandex.ru (S.V.); ladywaits@yandex.ru (T.V.); 8 Faculty of Dentistry, Kazan State Medical University, 420012 Kazan, Russia; rinat.saleev@gmail.com (R.S.); dr_ochnev001@mail.ru (G.S.); rin-gul@mail.ru (N.S.)

**Keywords:** COVID-19, healthcare workers, intensive care unit, employment, psycho-emotional states, occupational burnout, depersonalization, emotional exhaustion, occupational stress, aggression

## Abstract

Working in intensive care units (ICUs) is stressful and potentially leads to various psycho-emotional disorders. Today, this issue represents a serious concern to the healthcare sector and affects the quality of healthcare provided. This study aimed to assess and compare the psycho-emotional state in COVID-19 and non-COVID-19 hospitals’ ICU healthcare workers (HCWs). From January to July 2021, we conducted an anonymous cross-sectional web survey of ICU physicians and nurses (*N* = 1259) of various hospitals in a metropolis with a population of over 10 million people. The statistical distributions of non-COVID-19 ICU HCWs showed the following results: emotional exhaustion levels (low 14.6%, average 30.8%, and high 54.6%); depersonalization levels (low 11.6%, average 16.5%, and high 71.9%); and reduced personal accomplishment levels (low 23.5%, average 40.3%, and high 36.2%). The statistical distributions of COVID-19 ICU HCWs showed the following results: emotional exhaustion levels (low 16.5%, average 31.5%, and high 52%); depersonalization levels (low 7.4%, average 9.4%, and high 83.1%); and reduced personal accomplishment levels (low 25.4%, average 45.4%, and high 29.1%). This study found a strong correlation between emotional exhaustion, aggression, and depersonalization in non-COVID-19 ICU HCWs and also found a correlation between their age, aggression, emotional exhaustion, and occupational stress.

## 1. Introduction

The novel coronavirus disease (COVID-19) has become a pandemic, posing significant challenges for the global healthcare sector. Studies have shown that this pandemic led to a worldwide crisis and, as a result, had a significant adverse effect on the physical health and the psycho-emotional health of frontline healthcare workers (HCWs) [1,2,3].

Records of the World Health Organization (September 2021) showed over 224,511,226 registered cases of COVID-19 infection with over 4,627,540 confirmed deaths, and among those cases, over 7,158,248 cases with over 193,468 deaths in Russia [4].

Psycho-emotional state deviations represent an international and national challenge and can cause serious consequences. Since the beginning of the COVID-19 pandemic, ICU HCWs have experienced significant physical and emotional strain [5]. According to Van Mol et al. (2015) and Teixeria et al. (2013), working in ICUs is stressful and rapidly leads to burnout syndrome and other psycho-emotional disorders [5,6]. Furthermore, a published systematic review and meta-analysis conducted by Jain and Yuan (2020) proved that, since the beginning of the pandemic, HCWs of intensive care units (ICUs) have experienced high physical and emotional strain [7].

Employment duration plays a role in developing mental health deviations; in a narrative literature review, Rajkumar (2020) showed that working for a long time can lead to severe deviations that are not immediately obvious but require complex treatment. Unfortunately, these deviations can also affect the quality of care provided to patients [8].

In a multi-center mixed-methods study conducted by Mehta et al. (2021) with 58 ICU participants, the Maslach Burnout Inventory (MBI) showed the following results: emotional exhaustion (71.4%), depersonalization (53.6%), and reduced personal accomplishment (53.6%) [8]. Additionally, in a cross-sectional survey conducted by Hu et al. (2021), the MBI showed that 800 physicians (71.3% of all physicians) and 881 nurses (68.3% of all nurses) were deemed to be burnout [8]. All studies have shown that the primary risk factors of burnout are having more night shifts, fewer paid vacation days, a high patient death rate, and poor patient conditions [9,10].

Managing the mental deviations of HCWs in ICUs is essential and carrying this out is possible by implementing an evidence-based strategy, and this is possible only after a comprehensive evaluation of their psycho-emotional state. This assessment should depend on the duration of work with COVID-19 patients (employment duration in COVID-19 ICUs) and should include an adequate assessment of various elements that contribute to the development of depersonalization, reduced personal accomplishment, and emotional exhaustion. It should also examine the relationships between them.

This study aims to assess the psycho-emotional state of ICU HCWs (physicians and nurses) and determine its deviations (emotional exhaustion, depersonalization, reduced personal accomplishment), aggression (physical, verbal, indirect, and index of aggressive reactions), and occupational stress. The study also aims to assess and compare the contribution of various indicators to the development of emotional exhaustion, depersonalization, and reduced personal accomplishment among HCWs (physicians and nurses) of COVID-19 ICUs depending on their employment duration.

## 2. Materials and Methods

### 2.1. Research Design

We conducted a cross-sectional anonymous web survey on the psycho-emotional state of ICUs’ physicians and nurses with a structured questionnaire. This study has not undergone formal ethical review, as the survey was designed to be completely anonymous. Based on the local regulations, anonymous surveys do not require approval by a bioethics committee. All participants agreed to participate in this study and signed an online informed consent. The study was conducted under the principles of the Declaration of Helsinki.

### 2.2. Research Conditions and Respondents

Russian ICU HCWs (N = 1259, aged from 21 to 73 years (Mean = 36.28; SD = 12.03)) of various COVID-19 hospitals in a metropolis (with a population of over 10 million people) were interviewed to assess their psycho-emotional state in the period from January to July 2021. The questionnaire was distributed via email and Facebook through the Russian Federation of Anaesthesiologists and Reanimatologists (FAR) and other Russian medical communities. The general data on the respondents are presented in Table 1.

### 2.3. Questionnaire Composition

For respondents’ occupational burnout syndrome diagnosis, the Maslach Burnout Inventory (MBI) Questionnaire was adapted and standardized according to Vodopyanova [11]. It comprises the following scales:

- Emotional exhaustion, which is expressed by a loss of interest in others, life dissatisfaction, etc. The value ranges from 0 to 54 (max.). Low level (0 to 15), average level (16 to 24), and high level (25 and higher). The scale contains 9 questions (Cronbach’s α = 0.896);
- Depersonalization: the formality of performing occupational duties, lack of personal involvement and empathy, indifference, and emotional detachment. The value ranges from 0 to 30 (max.). Low level (0 to 5), average level (5 to 10), and high level (11 and higher). The scale contains 5 questions (Cronbach’s α = 0.896);
- Reduced personal accomplishment is expressed by a decrease in work motivation, an increase in negativity toward official duties, etc. The value ranges are from 0 to 48, where 48 is the maximum level of occupational success, and 0 is the maximum level of reduced personal accomplishment. Low level (37 and higher), average level (31 to 36), and high level (0 to 30). The scale contains 5 questions (Cronbach’s α = 0.896).

To assess aggressive and hostile reactions, the questionnaire of Bass and Darki (standardized by Hwang) was used [12], and it includes the following scales:

- Physical aggression is behavior causing or threatening physical harm toward others. The value ranges from 0 to 100 (max.). Low level (0 to 30), average level (31 to 52), advanced level (53 to 74), high level (75 to 85), and extremely high level (86 and higher). The scale contains 7 forward and 3 backward questions (Cronbach’s α = 0.422). Examples of statements: “I never get annoyed enough to throw things around” and “Anyone who insults me and my family asks for a fight”;
- Verbal aggression (quarrels, threats, etc.). The value ranges from 0 to 104 (max.). Low level (0 to 30), average level (31 to 52), advanced level (53 to 74), high level (75 to 85), and extremely high level (86 and higher). The scale contains 9 forward and 4 backward questions (Cronbach’s α = 0.648). Examples of statements: “If I disapprove of my friends’ behavior, I let them feel it” and “When they shout at me, I start shouting back”;
- Indirect aggression (directed indirectly toward others): gossip, hitting objects, shouting, or stamping your feet in a fit of rage. The value ranges from 0 to 99 (max.). Low level (0 to 14), average level (15 to 36), advanced level (37 to 58), high level (59 to 69), and extremely high level (70 and higher). The scale contains 6 forward and 3 backward questions (Cronbach’s α = 0.665). Examples of statements: “I am never gloomy with anger” and “When I get annoyed, I slam doors”.
- Physical aggression, indirect aggression, and verbal aggression together form a total index of aggressive behavior. The value ranges from 0 to 101.

The scale of occupational stress assessment of Weissman was used to diagnose the level of respondent’s occupational stress, which can be expressed in psychological and physiological reactions to a difficult work situation. The value ranges from 15 to 75 (max.). The scale contains 15 questions (Cronbach’s α = 0.824) [13].

The results were processed using IBM SPSS Statistical Software (v.26, IBM, Armonk, NY, USA) and Microsoft Excel Software Packages (v.2016, Microsoft, Redmond, WA, USA), and the following statistical tests were applied:

- Contingency tables (Pearson’s *χ*^2^ Test) were used to analyze the joint frequency distributions and assess their statistical reliability.
- Mann–Whitney U-criterion was used to assess the differences between two independent samples of respondents (HCWs) in COVID-19 and non-COVID-19 ICUs regarding the level of aggressive behavior, components of occupational burnout, and occupational stress.
- Correlation analysis of connections (Ro–Spearman) was used to study the connections between the studied signs.
- Multiple linear regression analysis was used to assess the size of the contribution of predictors to changes in the variance of dependent variables.
- The Kruskal–Wallace H-test was used to assess the differences between several independent samples of physicians and nurses with different employment experiences.
- Cluster analysis (k-means clustering) was used to determine the levels of occupational burnout in the sample.

## 3. Results

**Figure 1:**
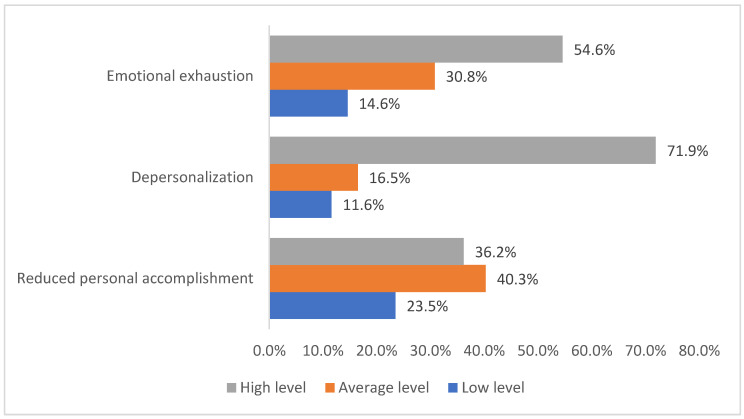
The frequency distribution of HCWs of non-COVID-ICU by the levels of severity of occupational burnout components.

The frequency distribution of HCWs of non-COVID-19 ICUs by the levels of severity of the components of occupational burnout (emotional exhaustion, depersonalization, and reduced personal accomplishment) is represented in Figure 1. Emotional exhaustion comprised 14.6% (54) low level, 30.8% (114) average level, and 54.6% (202) high level. Depersonalization comprised 11.6% (43) low level, 16.5% (61) average level, and 71.9% (266) high level. Reduced personal accomplishment comprised 23.5% (87) low level, 40.3% (149) average level, and 36.2% (134) high level.

**Figure 2:**
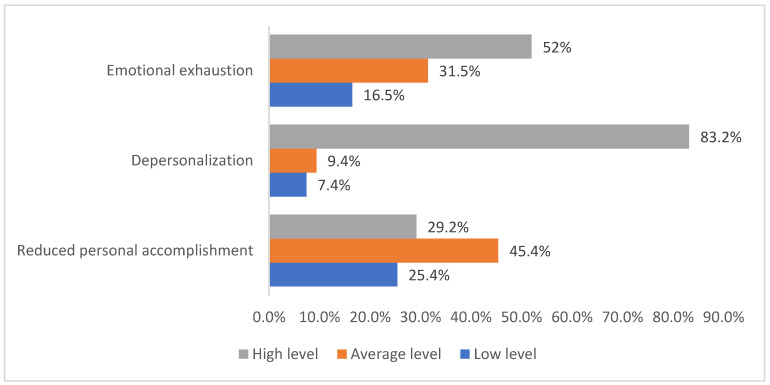
The frequency distribution of HCWs of COVID-19 ICU by the levels of severity of occupational burnout components.

The frequency distribution of HCWs of COVID-ICU by the levels of severity of the components of occupational burnout (emotional exhaustion, depersonalization, and reduced personal accomplishment) is represented in Figure 2. Emotional exhaustion comprised 16.5% (147) low level, 31.5% (280) average level, and 52% (462) high level. Depersonalization comprised 7.4% (66) low level, 9.4% (84) average level, and 83.1% (739) high level. Reduced personal accomplishment comprised 25.4% (226) low level, 45.4% (404) average level, and 29.1% (259) high level.

**Figure 3:**
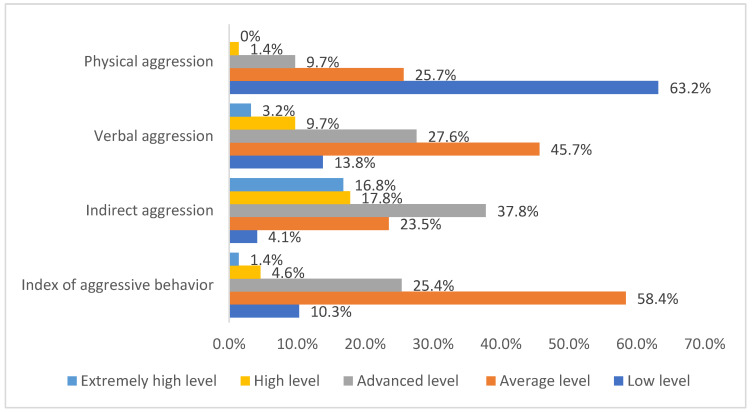
The frequency distribution of HCWs of non-COVID-19 ICUs by the levels of aggression and its components (physical, verbal, indirect, and index of aggressive behavior).

The frequency distribution of HCWs of non-COVID-19 ICUs by the levels of aggression and its components (physical aggression, verbal aggression, indirect aggression, and index of aggressive behavior) is represented in Figure 3. Physical aggression comprised 63.2% (234) low level, 25.7% (95) average level, 9.7% (36) advanced level, high level 1.4% (5), and 0% (0) extremely high level. Verbal aggression comprised 13.8% (51) low level, 45.7% (169) average level, 27.6% (102) advanced level, 9.7% (36) high level, and 3.2% (12) extremely high level. Indirect aggression comprised 4.1% (15) low level, 23.5% (87) average level, 37.8% (140) advanced level, 17.8% (66) high level, and 16.8% (62) extremely high level. The index of aggressive behavior comprised 10.3% (38) low level, 58.4% (216) average level, 25.4% (94) advanced level, 4.6% (17) high level, and 1.4% (5) extremely high level.

**Figure 4:**
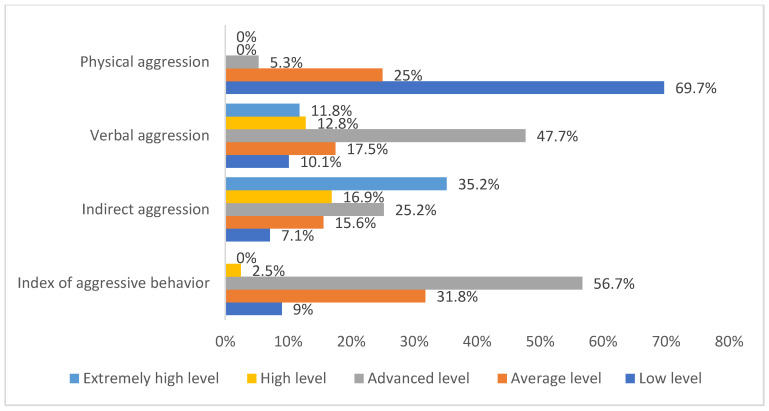
The frequency distribution of HCWs of COVID-19 ICUs by levels of aggression and its components.

The frequency distribution of HCWs of COVID-19 ICUs by the levels of aggression and its components (physical aggression, verbal aggression, indirect aggression, and index of aggressive behavior) is represented in Figure 4. Physical aggression comprised 69.7% (620) low level, 25% (222) average level, 5.3% (47) advanced level, 0% (0) high level, and 0% (0) extremely high level. Verbal aggression comprised 10.1% (90) low level, 17.5% (156) average level, 47.7% (424) advanced level, 12.8% (114) high level, and 11.8% (105) extremely high level. Indirect aggression comprised 7.1% (63) low level, 15.6% (139) average level, 25.2% (224) advanced level, 16.9% (150) high level, and 35.2% (313) extremely high level. The index of aggressive behavior comprised 9% (80) low level, 31.8% (283) average level, 56.7% (504) advanced level, 2.5% (22) high level, and 0% (0) extremely high level.

To avoid differences in the results, a random selection of 370 observations of the category “non-COVID-19 ICUs” from the variable “COVID-19 ICUs” was carried out to balance the comparison of categories because of the significant inequality among respondent groups working in different COVID-19 ICUs. The selection was carried out using IBM SPSS Statistical Software (v.26, IBM, Armonk, NY, USA). The obtained sample mean (N = 740) for statistical analysis comprised ICU HCWs aged 21 to 73 years (Mean = 35.58; SD = 11.36). The distribution of the general sample had a distinct character (Kolmogorov–Smirnov Criterion, *p* = 0.000), and based on that, statistical inference nonparametric methods were used as independent of the distribution.

Among COVID-19 and non-COVID-19 ICU HCWs, the Mann-Whitney U-test revealed significant differences in the severity of occupational stress and aggression with its components, such as physical, verbal, and indirect aggression (Mann-Whitney U-test, *p* = 0.000). It also revealed differences in the tendencies level of the severity of emotional exhaustion (U = 63,052,000; *p* = 0.063) and depersonalization (U = 62,966,000; *p* = 0.059). It found no differences for reduced personal accomplishment.

An analysis of the average values showed that occupational stress, depersonalization, and aggression with its components, such as indirect and verbal aggression, were higher among COVID-19 ICU HCWs. The level of physical aggression and emotional exhaustion was higher among non-COVID ICU HCWs (Table 2).

The results showed that among both COVID-19 and non-COVID-19 ICU HCWs (based on the methodology scales), a high level of emotional exhaustion and depersonalization, an average level of occupational stress and aggressive behavior, and an advanced level of indirect aggression. Among non-COVID-19 ICU HCWs (based on the methodology scales), physical and verbal aggression are at an average level, and among COVID-19 ICU HCWs (based on the methodology scales), physical aggression is at a low level, and verbal aggression is at an advanced level. In COVID-19 and non-COVID-19 ICU HCWs (based on the methodology scales), there is a high level of emotional stress, depersonalization, an average level of occupational stress and aggressive behavior, and an advanced level of indirect aggression.

To highlight the levels of occupational burnout in the sample, a cluster analysis (k-means clustering) identified two levels of occupational burnout (Table 3). The first cluster (N = 408), based on the methodology scales and the sample, included HCWs with average indicators of emotional exhaustion, depersonalization, and reduced personal accomplishment. The second cluster (N = 332), based on the methodology scales and the sample, included HCWs with high indicators of emotional exhaustion, depersonalization, and reduced personal accomplishment. Differences between clusters were significant in all parameters (Mann–Whitney U-test; *p*-value = 0.000). Thus, HCWs had an average and high level of occupational burnout.

The analyses of frequency distribution (using Pearson *χ*^2^ Statistics) of the occupational burnout (using K-Mean Clustering) of COVID ICUs HCWs are presented in Table 4.

Analyzing the joint frequency distributions (using Pearson’s *χ*^2^ statistics) showed significant differences:

(1) Significant differences were found in the level of aggressive behavior of COVID-19 ICUs HCWs (*χ*^2^ = 77.059; *p* = 0.000). The analysis showed that in non-COVID-19 ICUs, the number of HCWs with an average level of aggressive behavior was higher than expected, and the number of HCWs with an advanced level of aggressive behavior was lower than expected. In non-COVID-19 ICUs, the number of HCWs with an advanced level of aggressive behavior was higher than expected, and the number of HCWs with an average level of aggressive behavior was lower than expected. The number of HCWs with other levels of aggressive behavior in non-COVID-19 ICUs was almost non-existent. Not as expected, non-COVID-19 ICUs had more HCWs with an average level of aggressive behavior, and COVID-19 ICUs had more HCWs with an advanced level of aggressive behavior. Therefore, HCWs in non-COVID-19 ICU HCWs had an average level of aggressive behavior, while COVID-19 ICU HCWs had an advanced level of aggressive behavior.
(2) Significant differences were found in the level of physical aggression of HCWs in COVID-19 ICUs (*χ*^2^ = 9.843; *p* = 0.020). The analysis showed that in non-COVID-ICUs, the number of HCWs with a low level of physical aggression was lower than expected, and the number of HCWs with an advanced level of physical aggression was higher than expected. In COVID-19 ICUs, the number of HCWs with high levels of physical aggression was lower than expected, and the number of HCWs with low levels of physical aggression was higher than expected. Additionally, the number of HCWs with other levels of physical aggression in the COVID-19 and non-COVID-19 ICUs was the same as expected, and HCWs with an extremely high level of aggression were not found in the sample. Thus, HCWs in non-COVID-19 ICUs had an advanced level of physical aggression, and HCWs in COVID-19 ICUs had a low level of physical aggression. Both COVID-19 and non-COVID-19 ICUs had a larger number of HCWs with a low level of aggressive behavior, but in COVID-19 ICUs, this percentage was higher (70%). In non-COVID-19 ICUs, there was a larger percentage of HCWs with other levels of aggressive behavior.
(3) Significant differences were found in the level of verbal aggression of the HCWs of COVID-19 ICUs (*χ*^2^ = 82.676; *p* = 0.000). The analysis showed that in non-COVID-19 ICUs, the number of HCWs with low and average levels of verbal aggression was higher than expected, and the number of HCWs with advanced, high, and extremely high levels of verbal aggression was lower than expected. In COVID-19 ICUs, the number of HCWs with low and average levels of verbal aggression was lower than expected, and the number of HCWs with advanced, high, and extremely high levels of verbal aggression was higher than expected. Therefore, non-COVID-19 ICU HCWs had a low and average level of verbal aggression, while COVID-19 ICU HCWs had an advanced, high, and extremely high level of verbal aggression. Non-COVID-19 ICUs had a higher number of HCWs with an average level of verbal aggression, and COVID-19 ICUs had a higher number of HCWs with an advanced level of verbal aggression.
(4) Significant differences were found in the level of depersonalization of HCWs in COVID-19 ICUs (*χ*^2^ = 12.410; *p* = 0.002). The analysis showed that in non-COVID-19 ICUs, the number of HCWs with low and average levels of depersonalization was higher than expected, and the number of HCWs with high levels of depersonalization was lower than expected. In COVID-19 ICUs, the number of HCWs with low and average levels of depersonalization was lower than expected, and the number of HCWs with high levels of depersonalization was higher than expected. Therefore, HCWs in non-COVID-ICUs had a low and average level of depersonalization, and HCWs in COVID-19 ICUs had a high level of depersonalization. COVID-19 and non-COVID-19 ICUs had a larger number of HCWs with a high level of depersonalization, but in COVID-19 ICUs, this percentage was higher (61.6%), as well as the percentage of HCWs with an average level of depersonalization (30.5%). In non-COVID-19 ICUs, there was a higher percentage of HCWs with a low level of depersonalization (17%).
(5) No differences were found in the level of reduced personal accomplishment, emotional exhaustion, and occupational stress. In the analysis of indirect aggression, the same results were obtained as in the use of Mann–Whitney U-criterion (Table 2).

The results of a correlation analysis (Ro–Spearman) on a sample of non-COVID-19 ICU HCWs (N = 370) are presented in Table 5. They showed weak negative correlations at high levels of significance for the reduced personal accomplishment with occupational stress and indirect aggression. They also showed weak positive correlations on high levels of significance of occupational stress with emotional exhaustion, depersonalization, indirect and verbal aggression, aggressive behavior, emotional exhaustion with indirect aggression and aggressive behavior, depersonalization with indirect, verbal aggression, of aggressive behavior, and reduced personal accomplishment with indirect aggression.

Accordingly, among ICU HCWs: (1) A higher level of occupational stress resulted in a higher level of emotional exhaustion, depersonalization, personal achievement reduction, indirect, verbal aggression, and aggressive behavior; (2) A higher level of emotional exhaustion resulted in a higher level of indirect aggression and of aggressive behavior; (3) A higher level of depersonalization resulted in a higher level of indirect, verbal aggression and aggressive behavior. (4) A higher level of reduced personal accomplishment resulted in a higher level of indirect aggression.

The results of a correlation analysis (Ro–Spearman) on a sample of COVID-19 ICU HCWs (N = 370) are presented in Table 6.

Correlation analysis (Ro–Spearman) on a sample of COVID-19 ICU HCWs (N = 370) showed a weak negative correlation at high levels of significance for personal achievement reduction with occupational stress, physical and verbal aggression, and aggressive behavior, age with physical, indirect, and verbal aggression, and aggressive behavior, and employment duration in COVID-19 ICUs with physical and verbal aggression. It also showed aggressive behavior, with average negative correlations at high levels of significance of reduced personal accomplishment with indirect aggression. It also showed weak positive correlations at high levels of significance of age with reduced personal accomplishment, occupational stress with depersonalization, and indirect aggression, emotional exhaustion with physical, indirect aggression, and aggressive behavior, and depersonalization with physical aggression, as well as average positive correlations at high levels of occupational stress significance with emotional exhaustion.

Accordingly, among COVID-19 ICU HCWs: (1) A higher level of occupational stress resulted in a higher level of emotional exhaustion, depersonalization, reduced personal accomplishment, and indirect aggression; (2) A higher level of reduced personal accomplishment resulted in a higher level of physical, indirect, verbal aggression, and aggressive behavior; (3) A higher level of emotional exhaustion resulted in a higher level of physical, indirect aggression and aggressive behavior; (4) A higher level of depersonalization resulted in a higher level of physical aggression.

Moreover, with longer employment duration in COVID-19 ICUs, HCWs had a lower level of physical and verbal aggressio, and aggressive behavior, while older HCWs had a lower level of reduced personal accomplishment, physical, indirect, and verbal aggression, and aggressive behavior.

The parameters that contribute to the development of emotional exhaustion, depersonalization, and reduced personal accomplishment among non-COVID-19 ICUs HCWs and the results of multiple regression analyses are presented in Table 7, Table 8 and Table 9.

A multiple regression analysis (Table 7) showed that among non-COVID-19 ICU HCWs, there was a significant contribution of occupational stress and indirect aggression to emotional exhaustion. The high level of occupational stress and indirect aggression explain the high level of emotional exhaustion among non-COVID ICU HCWs (34.2%).

A multiple regression analysis (Table 8) showed that among non-COVID-19 ICU HCWs, there was a significant contribution of occupational stress and indirect aggression to the development of depersonalization. The high level of occupational stress and indirect aggression explain the high level of depersonalization among non-COVID-19 ICU HCWs (21.9%).

A multiple regression analysis (Table 9) showed that among non-COVID-19 ICU HCWs, there was a significant contribution of occupational stress and both indirect and verbal aggression to the development of reduced personal accomplishment. A high level of occupational stress and indirect aggression and a low level of verbal aggression explain the high level of reduced personal accomplishment among non-COVID-19 ICU HCWs (12.2%).

The parameters that contribute to developing emotional exhaustion and depersonalization among COVID-19 ICU HCWs were the highest in a multiple regression analysis, and those results are presented in Table 10 and Table 11.

A multiple regression analysis (Table 10) showed that among COVID-19 ICU HCWs, there was a significant contribution of occupational stress and aggression (indirect and physical) to the development of emotional exhaustion. The high level of occupational stress and aggression (indirect and physical) explains the high level of emotional exhaustion among COVID-19 ICU HCWs (32.5%).

A multiple regression analysis (Table 11) showed that among COVID-19 ICU HCWs, there was a significant contribution of occupational stress and aggression (indirect, verbal, and physical) to the development of depersonalization. The high level of occupational stress, verbal and physical aggression, and a low level of indirect aggression explain the high level of depersonalization among COVID-19 ICU HCWs (31.2%).

Using the Mann–Whitney U-test (Table 12), among non-COVID-19 ICU HCWs, significant differences were revealed in the severity of the level of reduced personal accomplishment and aggressive components (physical, verbal, indirect, and aggressive behavior) (Mann–Whitney U-test, *p*-value < 0.05), and no differences were found for occupational stress, emotional exhaustion, depersonalization, and physical aggression. The analysis of the average values allows us to say that the level of aggressive components (physical, verbal, indirect, and aggressive behavior) is higher among non-COVID-19 ICU nurses, and the level of reduced personal accomplishment is higher among non-COVID-19 ICU physicians.

Regardless of the job position, among non-COVID-ICU HCWs (based on the methodology scales), verbal aggression, aggressive behavior, and reduced personal accomplishment are at an average level, and indirect aggression is at an advanced level.

The Mann–Whitney U-test (Table 13) showed significant differences in the severity level of depersonalization, aggressive behavior, and aggression, and no differences were found for physical aggression, reduced personal accomplishment, emotional exhaustion, and occupational stress. It also showed that among COVID-19 ICU HCWs with different job positions, there were significant differences in the severity of the level of depersonalization and aggression with its components, such as verbal and direct aggression. Analysis of the average values (Table 13) showed that the level of the aggression components (indirect and verbal) was higher among COVID-19 ICU physicians, and the level of depersonalization was higher among COVID-19 ICU nurses (Mann–Whitney U-test, *p*-value < 0.05).

Among COVID-19 ICU physicians (based on the methodology scales), indirect aggression was at a high level, and the index of aggressive behavior was at an advanced level. Among COVID-19 ICU nurses (based on the methodology scales), indirect aggression was at an advanced level and the index of aggressive behavior at an average level. Regardless of the job position in COVID-19 ICUs (based on the methodology scales), COVID-19 ICU HCWs had an advanced level of verbal aggression and a high level of depersonalization.

An assessment was conducted to study the changes and the difference in the psycho-emotional state between physicians and nurses. The respondents’ sample (N = 889) aged 21 to 73 years was carried out (age = 36.97; SD = 13.04). The study included 63.6% males and 36.4% females.

The Kruskal–Wallis H-test showed that among COVID-19 ICU nurses with different employment durations in COVID-19 ICUs (Table 14), there was a significant difference in the severity levels of occupational stress, aggression with its components, such as physical, verbal, and indirect aggression, as well as the components of occupational burnout (emotional exhaustion, depersonalization, and reduced personal accomplishment) (Kruskal–Wallace H-test; *p*-value < 0.05).

An analysis of the average values showed that, based on the employment duration, the highest indicators of verbal, indirect aggression, and aggressive behavior were among COVID-19 ICU nurses with an employment duration of 1 to 4 months. The highest indicators of physical aggression, emotional exhaustion, and reduced personal accomplishment were among COVID-19 ICU nurses with an employment duration of 5 to 8 months. The highest indicators of occupational stress were in COVID-19 ICU nurses with an employment duration of 9 months to 1 year. The highest rates of depersonalization were among COVID-19 ICU nurses with an employment duration of over 1 year.

Besides, the lowest level indicators of occupational stress, emotional exhaustion, depersonalization, and reduced personal accomplishment were among COVID-19 ICU nurses with an employment duration of 1 to 4 months. The lowest indicators of the level of verbal aggression were in COVID-19 ICU nurses with an employment duration of 5 to 8 months. The lowest level indicators of physical, indirect aggression, and aggressive behavior were among COVID-19 ICU nurses with an employment duration of 9 months to 1 year.

**Figure 5:**
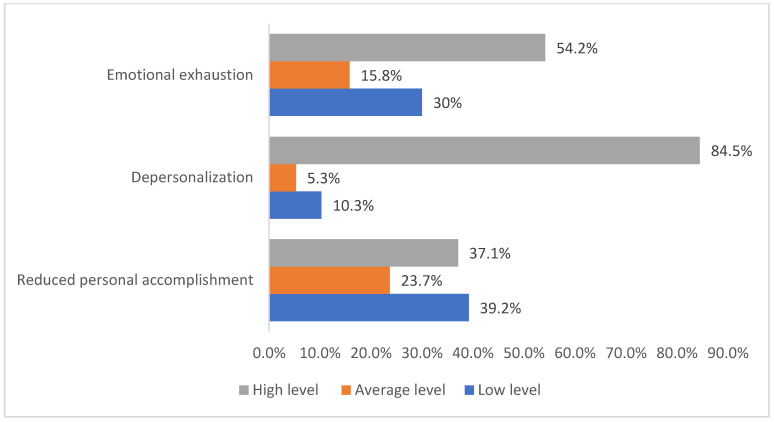
The frequency distribution of COVID-19 ICU nurses by the levels of severity of components of occupational burnout.

The frequency distribution of COVID-19 ICU nurses by the levels of severity of components of occupational burnout (emotional exhaustion, depersonalization, and reduced personal accomplishment) is represented in Figure 5. Emotional exhaustion comprised 30% (114) low level, 15.8% (60) average level, and 54.2% (206) high level. Depersonalization comprised 10.3% (39) low level, 5.3% (20) average level, and 84.5% (321) high level. Reduced personal accomplishment comprised 39.2% (149) low level, 23.7% (90) average level, and 37.1% (141) high level.

**Figure 6:**
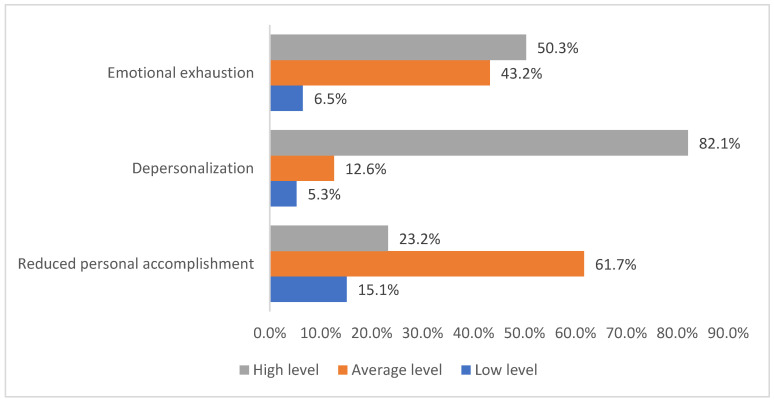
The frequency distribution of COVID-19 ICU physicians by the levels of severity of components of occupational burnout.

The frequency distribution of COVID-19 ICU physicians by the levels of severity of components of occupational burnout (emotional exhaustion, depersonalization, and reduced personal accomplishment) is represented in Figure 6. Emotional exhaustion comprised 6.5% (33) low level, 43.2% (220) average level, and 50.3% (256) high level. Depersonalization comprised 5.3% (27) low level, 12.6% (64) average level, and 82.1% (418) high level. Reduction of personal achievements comprised 15.1% (77) low level, 61.7% (314) average level, and 23.2% (118) high level.

Among COVID-19 ICU physicians with different employment durations, the Kruskal–Wallis H-test identified significant differences in the severity of occupational stress, aggression (physical, verbal, and indirect), and the components of occupational burnout, such as emotional exhaustion, depersonalization, and reduced personal accomplishment (Table 15).

An analysis of the average values showed that the highest levels of physical aggression were among COVID-19 ICU physicians with an employment duration of 1 to 4 months, and the highest indicators of verbal, indirect aggression, aggression, occupational stress, and depersonalization were among COVID-19 ICU physicians with an employment duration from 5 to 8 months. The highest rates of emotional exhaustion and reduction of personal achievements were among COVID-19 ICU physicians with an employment duration of 9 months to 1 year.

Besides, the lowest level indicators of occupational stress and reduced personal accomplishment were among COVID-19 ICU physicians with an employment duration of 1 to 4 months. The lowest level indicators of physical aggression were among COVID-19 ICU physicians with an employment duration of 5 to 8 months, and the lowest level indicators of verbal, indirect aggression, aggression, emotional exhaustion, and depersonalization were among COVID-19 ICU physicians with an employment duration of over one year (Table 15).

**Figure 7:**
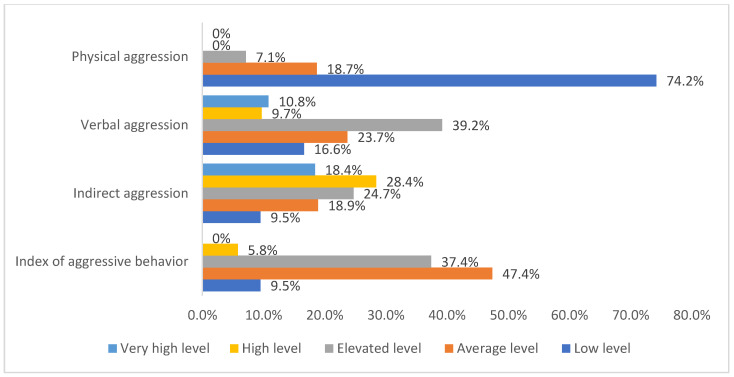
The frequency distribution of COVID-19 ICUs nurses by the levels of the components of aggression.

The frequency distribution of COVID-19 ICU nurses by the levels of the components of aggression (physical aggression, verbal aggression, indirect aggression, and index of aggressive behavior) is represented in Figure 7. Physical aggression comprised 74.2% (282) low level, 18.7% (71) average level, 7.1% (27) advanced level, 0% (0) high level, and 0% (0) extremely high level. Verbal aggression comprised 16.6% (63) low level, 23.7% (90) average level, 39.2% (149) advanced level, 9.7% (37) high level, and 10.8% (41) extremely high level. Indirect aggression comprised 9.5% (36) low level, 18.9% (72) average level, 24.7% (94) advanced level, 28.4% (108) high level, and 18.4% (70) extremely high level. Aggressive behavior comprised 9.5% (36) low level, 47.4% (180) average level, 37.4% (142) advanced level, 5.8% (22) high level, and 0% (0) extremely high level.

**Figure 8:**
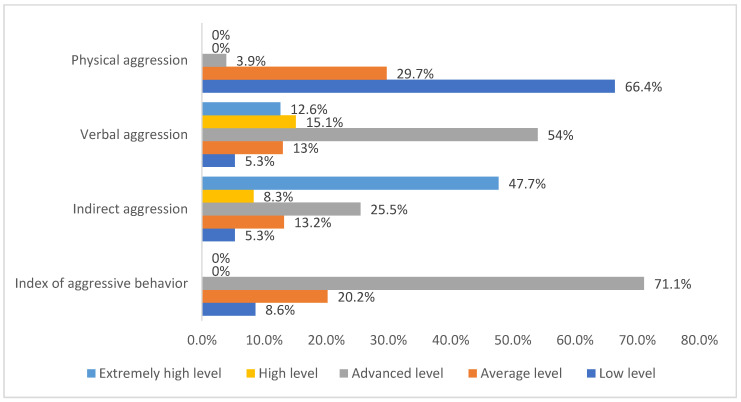
The frequency distribution of COVID-19 ICU physicians by the levels of the components of aggression.

The frequency distribution of COVID-19 ICU physicians by the levels of the components of aggression (physical aggression, verbal aggression, indirect aggression, and index of aggressive behavior) is represented in Figure 8. Physical aggression comprised 66.4% (338) low level, 29.7% (151) average level, 3.9% (20) advanced level, 0% (0) high level, and 0% (0) extremely high level. Verbal aggression comprised 5.3% (27) low level, 13% (66) average level, 54% (275) advanced level, 15.1% (77) high level, and 12.6% (64) extremely high level. Indirect aggression comprised 5.3% (27) low level, 13.2% (67) average level, 25.5% (130) advanced level, 8.3% (42) high level, and 47.7% (243) extremely high level. The aggressive behavior index comprised 8.6% (44) low level, 20.2% (103) average level, 71.1% (362) advanced level, 0% (0) high level, and 0% (0) extremely high level.

## 4. Discussion

Studies have shown that the SARS-CoV-2 pandemic led to an increase in cases of mental disorders among medical personnel, especially in intensive care units (ICUs) [14,15,16]. Guirardello (2017) proved there is a relationship between employment conditions in the ICU and the psycho-emotional state of its physicians and nurses (healthcare workers) [17]. Vasconcelos et al. showed that the work schedule and the number of days off play a significant role in increasing psycho-emotional deviations among the healthcare workers (HCWs) of the ICU [18]. Considering the studies of Guirardello and Vasconcelos et al., the results of our study (using Spearman’s Rank Correlation Coefficient) showed that employment duration has a significant effect on the psycho-emotional state of HCWs.

The Spearman’s Rank Correlation Coefficient showed that, among nurses, there was a weak negative correlation at high levels of significance of the employment durations in COVID-19 ICUs with physical aggression (Ro = −0.305; *p*-value < 0.01), indirect aggression (Ro = −0.235; *p*-value < 0.01), and aggressive behavior (Ro = −0.285; *p*-value < 0.01). Similarly, among physicians, there was a weak negative correlation at high levels of significance of the employment durations in COVID-19 ICUs with aggressive behavior (Ro = −0.202; *p*-value < 0.01), occupational stress (Ro = 0.242; *p*-value < 0.01), and reduced personal accomplishment (reverse scale) (Ro = −0.238; *p*-value < 0.01). Based on that, with a longer employment duration in COVID-19 ICUs, nurses experienced a lower level of aggression (physical and indirect) and aggressive behavior. Additionally, with a longer employment duration in COVID-19 ICUs, physicians experienced a lower level of aggressive behavior, a higher level of occupational stress, and reduced personal accomplishment.

The results of a systematic review conducted by Rotenstein et al. showed that 80% of HCWs are subject to various deviations of the psycho-emotional state (burnout syndrome, depersonalization, and reduced personal accomplishment) [19]. According to Galehdar et al., the specificities of working with COVID-19 infected patients in the ICU, such as wearing personal protective equipment (PPE) and direct contact with infected patients with a severe condition, can lead to an increase in many manifestations, including but not limited to burnout syndrome, increased anxiety, and emotional exhaustion [20]. The results of our study showed that physicians and nurses in the COVID-ICU had similar deviations of the psycho-emotional state.

According to a systematic review and meta-analyses conducted by Pappa et al., which included thirteen studies with 33,062 participants, anxiety and depression were assessed in 12 studies, with a pooled prevalence of 23.2% and depression in 10 studies, with a prevalence rate of 22.8%. This data supports the previously published online survey conducted by Wang et al. [21,22].

A systematic literature review conducted by Van Mol et al. (2015) analyzed publications that included 14,770 respondents and showed that, in the ICU, the reported prevalence of burnout is up to 70.1%, depersonalization is up to 41.8%, and emotional exhaustion is up to 52%, and this data supports the previously published online survey conducted by Barbosa et al. [6,23].

In a cross-sectional study conducted by Fernández-Prada et al., with a sample of 42 junior medical physicians who are on duty in the emergency department, 45% of physicians revealed emotional exhaustion and a high depersonalization value, simultaneously with a high level of aggression of 38% [24]. Elbay et al. (2020) conducted an online survey with 442 participants (healthcare workers), which showed supporting data; 64.7% had burnout syndrome, and 41.2% had an increased stress level [16].

Studies have shown that the levels of occupational stress, depersonalization, and aggressive behavior are higher among COVID-19 ICU HCWs, and the level of physical aggression and emotional exhaustion are higher among non-COVID-19 ICUs [25,26,27,28]. The results of those studies are partially consistent with the results of our study.

Interestingly, most HCWs in COVID-19 hospitals had an average level of occupational burnout because of their gradual adaptation to working conditions. Trumello et al. (2020) and Rosted et al. (2021) showed a greater manifestation of HCWs in COVID-19 hospitals, with direct dependence on the number of patients [1,29].

According to a study conducted by Abdelhafiz et al. (2020), the primary risk factors for burnout syndrome development are the risk of infection, young age, and the purchase of PPE [30]. However, in an observational study (*n* = 1961), Lasalvia et al. (2021) revealed that 38% of the HCWs of COVID-19 hospitals are subject to burnout syndrome corresponding to those obtained in this work, which leaves this question open and requires a longitudinal study [31]. The gradual adaptation to working conditions of most COVID-19 ICU HCWs explains their experience with an average level of occupational burnout.

According to previous studies, among non-COVID ICU HCWs: (1) A higher level of occupational stress resulted in a higher level of emotional exhaustion, depersonalization, reduced personal accomplishment, indirect aggression, verbal aggression, and aggressive behavior; (2) A higher level of emotional exhaustion resulted in a higher level of indirect aggression and aggressive behavior; (3) A higher level of depersonalization resulted in a higher level of indirect aggression, verbal aggression, and aggressive behavior; (4) A higher level of reduced personal accomplishment resulted in a higher level of indirect aggression. The data obtained in the literature is consistent with the data of our study [30,31,32,33].

Other previous studies showed that among COVID-19 ICU HCWs: (1) A higher level of occupational stress resulted in a higher level of emotional exhaustion, depersonalization, reduced personal accomplishment, indirect, verbal aggression, and aggressive behavior; (2) A higher level of emotional exhaustion resulted in a higher level of indirect aggression and aggressive behavior; (3) A higher level of depersonalization resulted in a higher level of indirect aggression, verbal aggression, and aggressive behavior; (4) A higher level of reduced personal accomplishment resulted in a higher level of indirect aggression. The data obtained in our study are consistent with those obtained earlier in other studies [31,32,33,34].

It is essential to mention that the primary goal of psycho-emotional health therapy is managing occupational stress, emotional exhaustion, and reduced personal accomplishment. West et al. proved that these elements have a significant impact on the quality of care provided to the patient [35].

Psycho-emotional deviations lead to a decrease in the department’s efficiency [36]. In a literature review, Romani and Ashkar discussed the efficiency of combined work with a psychologist in managing those deviations [37]. The hypothesis of Torres et al. showed that four therapy sessions had positive results [38]. Because of its gradual decrease, it is possible to postpone the management of aggressive behavior among COVID-19 ICU HCWs.

It is essential to clarify changes in the psycho-emotional state of HCWs, including physicians and nurses, based on their work length in the ICUs of COVID-19 hospitals, and it is also vital to prevent the development of any psycho-emotional deviations. COVID-19 ICU nurses can be affected by various forms of aggression, emotional exhaustion, and reduced personal accomplishment, which can reach its greatest severity with an employment duration of up to 8 months, decreasing with an increase in the employment duration, while depersonalization increases. The results of our study showed that nurses with an employment duration of up to 8 months are like those of Jose et al. [39].

A systematic review conducted by Edward et al. (2014) examined occupational anxiety related to actual aggression in the workplace among nurses and showed that the unwillingness to work in hospitals’ ICU can lead to the manifestation of emotional burnout, depersonalization, and consequently to a decrease in the quality of care provided [40].

According to Giménez-Espert et al., risk factors for the development of these conditions are the serious condition of patients, the wearing of PPE, the difficult epidemiological situation in the region, lack of resources, etc. [41]. Thus, the nurses working in COVID-19 ICUs, with longer employment duration, had a lower level of physical, indirect aggression, and aggressive behavior in general.

All indicators reach a maximum with an employment duration of up to one year and gradually decrease with an increase in the employment duration of physicians. This data differs from the results of Elghazally et al. but is consistent with the data of Firew et al., and this is because of gradual adaptation to the load, as well as improvement of the treatment methods of COVID-19 infected patients [42,43].

According to a study published by Miguel-Puga, these deviations can lead to the development of post-traumatic syndrome, which potentially reduces the quality of care provided [44]. According to Sharma et al., the risk of infection of close people, wearing PPE, low communication with superiors and colleagues, and the severe conditions of patients are risk factors for the development of listed conditions [45].

Therefore, physicians working in COVID-19 ICUs had a lower level of aggressive behavior and a higher level of occupational stress and reduced personal accomplishment during longer employment.

The results of an advanced search using three bibliographic databases (PubMed, Google Scholar, and eLibrary) showed that this study is one of the first to identify the effect of the duration of working time of HCWs (physicians and nurses) in COVID-19 hospitals and the deviations of their psycho-emotional state. While conducting the study, we did not consider the number of patients and the severity of their conditions, the gender of the respondents, the work schedule of the respondent, the relationship of the respondents with colleagues in the working environment, the total work experience, and the vacation policies.

## 5. Conclusions

In this study, we assessed the psycho-emotional state of healthcare workers (HCWs) of COVID-19 and non-COVID-19 intensive care units (ICUs). Results of the study showed that COVID-19 ICU HCWs had a higher level of occupational stress, depersonalization, and aggression (indirect and verbal aggression) than non-COVID-19 ICU HCWs. Non-COVID-19 ICU HCWs experienced a higher degree of physical aggression and emotional exhaustion than COVID-19 ICU HCWs.

Occupational stress had the highest level of psycho-emotional state deviation development among non-COVID-19 ICU HCWs. However, employment duration, age, reduced personal accomplishment, and occupational stress affect the psycho-emotional state of COVID-19 ICU HCWs, and among all those elements, age and occupational stress had the highest effects.

COVID-19 ICU nurses experienced various forms of aggression, emotional exhaustion, and reduced personal accomplishment that reached their greatest severity with up to 8 months of employment and then decreased with a long employment duration, while depersonalization increased.

COVID-19 ICU physicians experienced emotional exhaustion, depersonalization, reduced personal accomplishment, physical, verbal, and indirect aggression, aggressive behavior, and occupational stress. Those deviations reached their maximum level with up to one year of employment and gradually decreased with an increase in employment duration.

In the future, these results can contribute to the development of complex psychotherapy for ICU HCWs of COVID-19 and non-COVID-19 hospitals to maintain the quality of care provided to patients at a high level. Understanding the gradation of the psycho-emotional deviations based on the employment terms in COVID-19 ICUs can help develop a step-by-step therapeutic management strategy and achieve its values.

## Figures and Tables

**Table 1:** General information about the respondents.

Characteristic	Number (%) of Respondents
Male	684 (54.3%)
Female	575 (45.7%)
Employment in COVID-19 ICUs	
Employed	889 (70.6%)
Non-employed	370 (29.4%)
Duration of work (employment) in COVID-19 ICUs at the time of the survey	
More than 1 year	124 (9.8%)
11 months–1 year	128 (10.2%)
9–10 months	154 (12.2%)
7–8 months	136 (10.8%)
5–6 months	152 (12.1%)
3–4 months	197 (15.6%)
1–2 months	65 (5.2%)
Do not work in COVID-19 ICUs	303 (24.1%)
Job position in the ICU	
Physician	767 (60.9%)
Nurse	492 (39.1%)
Duration of work in the specified job position (the specialty)	
More than 8 years	535 (42.5%)
5–7 years	196 (15.6%)
2–4 years	171 (13.6%)
6 months–1 year	232 (18.4%)
4–5 months	95 (7.5%)
1–3 months	30 (2.4%)
Specialty in the COVID-19 ICU at the time of the survey	
Practice their specialty	864 (68.6%)
Were retrained for another specialty	34 (2.7%)
Do not practice their specialty	358 (28.7%)

ICUs are the intensive care units.

**Table 2:** Assessment of the level of aggression (physical, indirect, and verbal), aggressive behavior, occupational stress, emotional exhaustion, and depersonalization in ICU HCWs.

Scale	Non-COVID-19 Hospitals	COVID-19 Hospitals	Reliability of Differences
Physical aggression	M = 31.62 SD = 17.990	M = 27.16 SD = 16.307	U = 58,435.000 *p* = 0.000
Indirect aggression	M = 51.55 SD = 20.340	M = 58.51 SD = 24.686	U = 55,849.500 *p* = 0.000
Verbal aggression	M = 49.54 SD = 20.340	M = 61.92 SD = 21.166	U = 44,937.000 *p* = 0.000
Index of aggressive behavior	M = 44.2360 SD = 14.325	M = 49.1982 SD = 15.532	U = 50,431.500 *p* = 0.000
Occupational stress	M = 40.62 SD = 8.595	M = 42.91 SD = 8.889	U = 55,097.500 *p* = 0.000
Emotional exhaustion	M = 27.80 SD = 11.288	M = 26.59 SD = 10.055	U = 63,052.000 *p* = 0.063
Depersonalization	M = 13.35 SD = 5.775	M = 14.34 SD = 5.461	U = 62,966.000 *p* = 0.059

M is the mean value, SD is the standard deviation, U is the Mann–Whitney Criterion, and *p* is the significance level.

**Table 3:** Levels of occupational burnout among ICU HCWs (K-Means Clustering).

Scale	Occupational Burnout		Validity of Differences	
	Average Level	High Level	Mann-Whitney U-Test	*p*-Value
Emotional exhaustion	19	37	601.000	0.000
Depersonalization	11	17	22,068.000	0.000
Reduced personal accomplishment (reverse scale)	35	29	33,118.500	0.000
Number	408	332		

**Table 4:** The relationship between ICU HCWs’ occupational burnout and the profile of hospital admissions.

			Occupational Burnout	
			Average Level	High Level
**Profile of hospital admissions**	**Non-COVDI-19 hospitals**	**Actual frequency**	190	180
		**Expected frequency**	204	166
	**COVID-19 hospitals**	**Actual frequency**	218	152
		**Expected frequency**	204	166
*χ*^2^ = 4283, *p* = 0.038				

**Table 5:** Correlation analysis (Ro–Spearman) of occupational burnout, aggression, and occupational stress in non-COVID-19 ICU HCWs.

Scale	Emotional Exhaustion	Depersonalization	Reduced Personal Accomplishment (Reverse)	Indirect Aggression	Verbal Aggression	Index of Aggressive Behavior
Occupational stress	Ro = 0.486 **	Ro = 0.413 **	Ro = −0.255 **	Ro = 0.391 **	Ro = 0.269 **	Ro = 0.312 **
Emotional exhaustion	-	-	-	Ro = 0.325 **	-	Ro = 0.272 **
Depersonalization	-	-	-	Ro = 0.301 **	Ro = 0.246 **	Ro = 0.301 **
Reduced personal accomplishment (reverse)	-	-	-	Ro = −0.216 **	-	-

Ro is Spearman’s Criterion. ** is when *p*-value < 0.01.

**Table 6:** Correlation analysis (Ro–Spearman) of occupational burnout, aggressive behavior, occupational stress, and age and employment duration in COVID-19 ICU HCWs.

Scale	Emotional Exhaustion	Depersonalization	Reduced Personal Accomplishment (Reverse)	Physical Aggression	Indirect Aggression	Verbal Aggression	Index of Aggressive Behavior
Employment duration	-	-	-	Ro = −0.224 **	-	Ro = −0.259 **	Ro = −0.323 **
Age	-	-	Ro = 0.204 **	Ro = −0.257 **	Ro = −0.427 **	Ro = −0.232 **	Ro = −0.398 **
Occupational stress	Ro = 0.539 **	Ro = 0.404 **	Ro = −0.331 **	−	Ro = 0.327 **	-	-
Emotional exhaustion	-	-	-	Ro = 0.218 **	Ro = 0.395 **	-	Ro = 0.341 **
Depersonalization	-	-	-	Ro = 0.330 **	-	-	-
Reduced personal accomplishment (reverse)	-	-	-	Ro = −0.225 **	Ro = −0.515 **	Ro = −0.331 **	Ro = −0.483 **

Ro is Spearman’s Criterion. ** is when *p*-value < 0.01.

**Table 7:** Assessment of the contribution of occupational stress and indirect aggression to the development of emotional exhaustion in non-COVID-19 ICU HCWs.

Scale		R-Squared	F-Criteria	Durbin–Watson Criteria
Constant	β = −3.316 *p* = 0.153	0.342	F = 95.517 *p* = 0.000	1.978
Occupational stress	β = 0.665 *p* = 0.000			
Indirect aggression	β = 0.080 *p* = 0.003			

**Table 8:** Assessment of the contribution of occupational stress and indirect aggression to the development of depersonalization in non-COVID-19 ICU HCWs.

Scale		R-Squared	F-Criteria	Durbin–Watson Criteria
Constant	β = 0.662 *p* = 0.609	0.219	F = 51.548 *p* = 0.000	1.992
Occupational stress	β = 0.265 *p* = 0.000			
Indirect aggression	β = 0.037 *p* = 0.012			

**Table 9:** Assessment of the contribution of occupational stress and indirect and verbal aggression to the development of reduced personal accomplishment in non-COVID-19 ICU HCWs.

Scale		R-Squared	F-Criteria	Durbin–Watson Criteria
Constant	β = 41.436 *p* = 0.000	0.122	F = 16.935 *p* = 0.000	2.360
Occupational stress	β = −0.197 *p* = 0.000			
Indirect aggression	β = −0.059 *p* = 0.001			
Verbal aggression	β = 0.035 *p* = 0.042			

**Table 10:** Assessment of the contribution of occupational stress and aggression (indirect and physical) to the development of emotional exhaustion in COVID-19 ICU HCWs.

Scale		R-Squared	F-Criteria	Durbin–Watson Criteria
Constant	β = −0.804 *p* = 0.714	0.325	F = 58.874 *p* = 0.000	2.439
Occupational stress	β = 0.466 *p* = 0.000			
Indirect aggression	β = 0.086 *p* = 0.000			
Physical aggression	β = 0.088 *p* = 0.001			

**Table 11:** Assessment of the contribution of occupational stress and aggression (physical, indirect, and verbal) to the development of depersonalization in COVID-19 ICU HCWs.

Scale		R-Squared	F-Criteria	Durbin–Watson Criteria
Constant	β = 0.983 *p* = 0.421	0.312	F = 41.305 *p* = 0.000	1.968
Occupational stress	β = 0.262 *p* = 0.000			
Physical aggression	β = 0.098 *p* = 0.000			
Indirect aggression	β = −0.040 *p* = 0.000			
Verbal aggression	β = 0.029 *p* = 0.0047			

**Table 12:** Assessment of the average values of the level of reduction of personal achievements, components of aggression (physical, verbal, indirect, aggressive behavior) in non-COVID-19 ICU HCWs. M is the mean value, SD is the standard deviation, U is the Mann–Whitney criterion, and *p* is the significance level.

Scale	Nurses	Physicians	Reliability of Differences
Verbal aggression	M = 52.43 SD = 25.112	M = 48.28 SD = 17.789	U = 12,584.500 *p* = 0.047
Indirect aggression	M = 55.88 SD = 16.973	M = 49.67 SD = 21.397	U = 11,159.500 *p* = 0.002
Index of aggressive behavior	M = 47.32 SD = 14.418	M = 42.9 SD = 14.103	U = 11,159.500 *p* = 0.001
Reduced personal accomplishment (reverse scale)	M = 33.09 SD = 6.087	M = 31.71 SD = 6.728	U = 12,296.000 *p* = 0.022

**Table 13:** Assessment of the average values of aggression (verbal and indirect), aggressive behavior, and depersonalization in COVID-19 ICU HCWs. M is the mean value, SD is the standard deviation, U is the Mann–Whitney criterion, and p is the significance level.

Scale	Nurses	Physicians	Reliability of Differences
Verbal aggression	M = 59.58 SD = 23.820	M = 64.66 SD = 17.912	U = 88,855.500 *p* = 0.036
Indirect aggression	M = 52.51 SD = 20.641	M = 64.03 SD = 25.653	U = 68,847.000 *p* = 0.000
Index of aggressive behavior	M = 46.22 SD = 16.582	M = 52.16 SD = 13.71	U = 72,983.000 *p* = 0.000
Depersonalization	M = 15.17 SD = 6.307	M = 13.93 SD = 4.800	U = 78,898.500 *p* = 0.000

**Table 14:** Assessment of the average values of the levels of occupational stress, components of occupational burnout, components of aggression, and aggressive behavior in COVID-19 ICU nurses with different employment durations. M is the mean value, SD is the standard deviation, H is the Kruskal–Wallace Criterion, and p is the significance level.

Scale	1–4 Months	5–8 Months	9 Months–1 Year	Over 1 Year	Reliability of Differences
Physical aggression	M = 31.14 SD = 12.194	M = 36.76 SD = 20.449	M = 17.77 SD = 13.508	M = 22.31 SD = 11.724	H = 71.484 *p* = 0.000
Verbal aggression	M = 80.20 SD = 11.456	M = 45.33 SD = 24.647	M = 57.65 SD = 24.721	M = 60.31 SD = 14.106	H = 94.789 *p* = 0.000
Indirect aggression	M = 62.80 SD = 13.661	M = 56.40 SD = 18.359	M = 44.36 SD = 24.757	M = 49.64 SD = 16.416	H = 43.779 *p* = 0.000
Index of aggressive behavior	M = 58.05 SD = 7.599	M = 46.17 SD = 16.673	M = 39.93 SD = 19.125	M = 44.09 SD = 12.241	H = 60.056 *p* = 0.000
Occupational stress	M = 40.94 SD = 2.866	M = 41.71 SD = 10.808	M = 43.79 SD = 11.739	M = 42.85 SD = 8.751	H = 10.104 *p* = 0.018
Emotional exhaustion	M = 22.53 SD = 5.597	M = 32.00 SD = 11.471	M = 26.19 SD = 11.259	M = 24.23 SD = 11.034	H = 34.789 *p* = 0.000
Depersonalization	M = 13.61 SD = 1.690	M = 15.92 SD = 9.186	M = 14.82 SD = 5.532	M = 16.33 SD = 5.448	H = 17.153 *p* = 0.001
Reduced personal accomplishment (reverse scale)	M = 33.19 SD = 4.000	M = 27.88 SD = 12.408	M = 30.86 SD = 9.727	M = 33.05 SD = 6.212	H = 10.111 *p* = 0.018

**Table 15:** Assessment of the average values of occupational stress level, components of occupational burnout, components of aggression, and aggressive behavior in COVID-19 ICU physicians with different employment durations. M is the mean value, SD is the standard deviation, H is the Kruskal–Wallace Criterion, and p is the significance level.

Scale	1–4 Months	5–8 Months	9 Months–1 Year	More Than 1 Year	Reliability of Differences
Physical aggression	M = 31.27 SD = 20.513	M = 25.70 SD = 13.588	M = 27.31 SD = 12.616	M = 28.54 SD = 9.890	H = 7.960 *p* = 0.047
Verbal aggression	M = 62.98 SD = 25.257	M = 67.91 SD = 16.326	M = 65.23 SD = 8.336	M = 52.88 SD = 19.422	H = 30.686 *p* = 0.000
Indirect aggression	M = 55.00 SD = 33.161	M = 72.33 SD = 14.988	M = 68.12 SD = 22.771	M = 38.63 SD = 23.863	H = 58.141 *p* = 0.000
Index of aggressive behavior	M = 49.75 SD = 21.311	M = 55.31 SD = 6.707	M = 53.55 SD = 8.736	M = 40.02 SD = 15.466	H = 29.935 *p* = 0.000
Occupational stress	M = 39.30 SD = 6.436	M = 45.80 SD = 9.164	M = 45.23 SD = 8.039	M = 41.78 SD = 4.333	H = 64.529 *p* = 0.000
Emotional exhaustion	M = 26.39 SD = 12.917	M = 26.26 SD = 8.854	M = 29.91 SD = 5.401	M = 20.29 SD = 6.535	H = 76.483 *p* = 0.000
Depersonalization	M = 11.47 SD = 4.768	M = 15.30 SD = 4.725	M = 15.03 SD = 4.213	M = 11.05 SD = 2.915	H = 63.588 *p* = 0.000
Reduced personal accomplishment (reverse scale)	M = 35.65 SD = 4.713	M = 31.80 SD = 4.157	M = 28.82 SD = 4.469	M = 32.76 SD = 3.967	H = 111.989 *p* = 0.000

## Data Availability

Data is available upon request.
